# Gall Stone Ileus and Recurrence: Management Dilemma for the Operating Surgeon

**DOI:** 10.7759/cureus.75577

**Published:** 2024-12-12

**Authors:** A Samee, R Shete, S Rana, M Samee, Y Zubair, A Samee

**Affiliations:** 1 Department of Surgery, Royal Oldham Hospital, Northern Care Alliance NHS Trust, Manchester, GBR; 2 Department of Emergency Medicine, Royal Stoke University Hospital, Stoke On Trent, GBR; 3 Department of Radiology, Brighton and Sussex University Hospitals NHS Trust, Brighton, GBR; 4 Department of Acute Medicine, Royal Stoke University Hospital, Stoke On Trent, GBR

**Keywords:** cholecystocholedochal fistula, common enterotomy, diagnostic laparoscopy, duodenal fistula, frail elderly, frail patients, gallstone ileus, paralytic ileus, recurrent gall stone ileus, small bowel enterotomy

## Abstract

Gallstone ileus (GSI) is a rare complication of gallstone disease. It occurs as a result of the passage of a stone from the biliary tract into the gastrointestinal tract via an abnormal pathway (bilio-enteric fistula). Chronic inflammatory processes result in gall bladder adhering and subsequently eroding into the intestines, leading to a fistula. It is a surgical emergency seen in the elderly who often present as being unwell, with abdominal pain, distension, and vomiting. Imaging such as a CT scan is diagnostic in confirming small bowel obstruction. Management usually involves relief of obstruction by removing the impacted gallstone.

Despite surgical intervention, a small proportion of patients develop recurrent symptoms or recurrent gallstone ileus, usually within a few weeks of initial presentation. The recurrence of symptoms during index admission is extremely rare and can be challenging to diagnose and manage. The morbidity and mortality remain high in elderly patients.

We report an 89-year-old patient who presented with small bowel obstruction. The CT scan confirmed gallstone ileus as a result of a stone impacted in the mid-small bowel. The patient had a laparotomy with extraction of the stone. No migrating stones were felt proximally. Five days postoperatively, he developed recurrent gallstone ileus confirmed on a CT scan and had to undergo another surgery to relieve the obstruction.

We aim to investigate various management strategies for recurrent gallstone ileus, ranging from the commonly practiced approach of simple stone extraction to more definitive surgical interventions, including fistula repair, which may provide a more comprehensive solution.

## Introduction

Cholelithiasis is a common pathology affecting 10-15% of the population in developed countries [1]. Gallstone ileus (GSI) is a rare but serious complication affecting 0.5 to 1.5% of patients with gallstones and accounts for 0.1% of cases of mechanical bowel obstruction [2,3]. It occurs when a gallstone erodes through the biliary tract into the gastrointestinal tract via an abnormal communication known as a bilio-enteric fistula. The stones may pass naturally or get impacted, usually in the terminal ileum, resulting in mechanical obstruction. Management usually involves relief of obstruction by removing the obstructing gallstone via surgical intervention.

Considering the acute presentation and coexisting morbidities, the decision to perform the minimum required intervention, enterolithotomy, is often taken. While the majority of patients remain asymptomatic following relief from the bowel obstruction, about 5-20% of patients present with recurrent symptoms, i.e., recurrent gallstone ileus, usually within 6-9 months from the initial presentation [4-7]. The surgeon faces an operating dilemma: whether to proceed with surgery with curative intent (i.e., perform a comprehensive curative repair of the fistula) or opt for a less invasive procedure involving simple stone extraction (enterolithotomy) while accepting a small risk of a recurrence.

## Case presentation

An 89-year-old gentleman was brought in from a nursing home to the emergency department with abdominal pain, vomiting, and being 'off legs.' His past medical history included vascular dementia, hypertension, and a previous stroke.

On examination, the pulse was 110 beats per minute, blood pressure was 100/80 mm Hg, and respiratory rate was 18 per minute. The abdomen was soft, visibly distended, and mildly tender on deep palpation. There were no signs of peritonism. There were no lumps or masses to feel. Per rectal (PR) examination was normal. The blood results on arrival showed a raised white cell count of 12.5 (4.0-11.0 × 109/L), and C-reactive Protein (CRP) was 75 mg/L. The liver functions and renal functions were normal.

**Figure 1:**
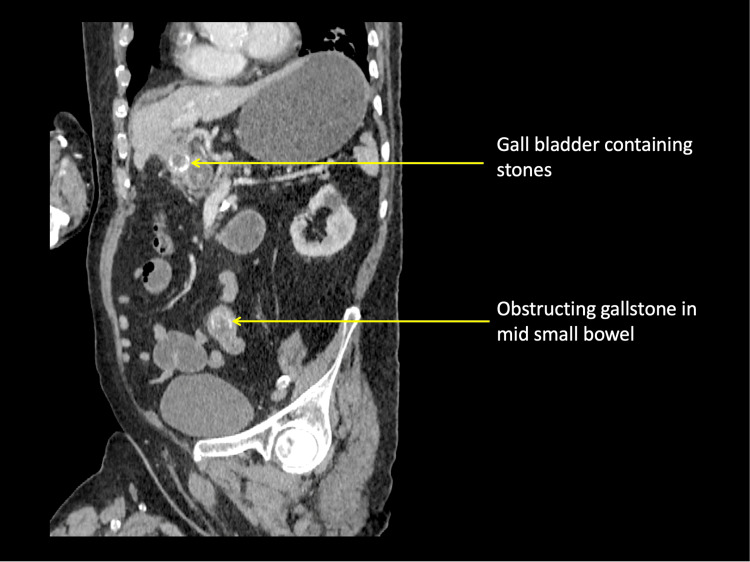
Coronal CT showing stones in the gall bladder. An obstructing gall stone in mid small bowel is noted.

**Figure 2:**
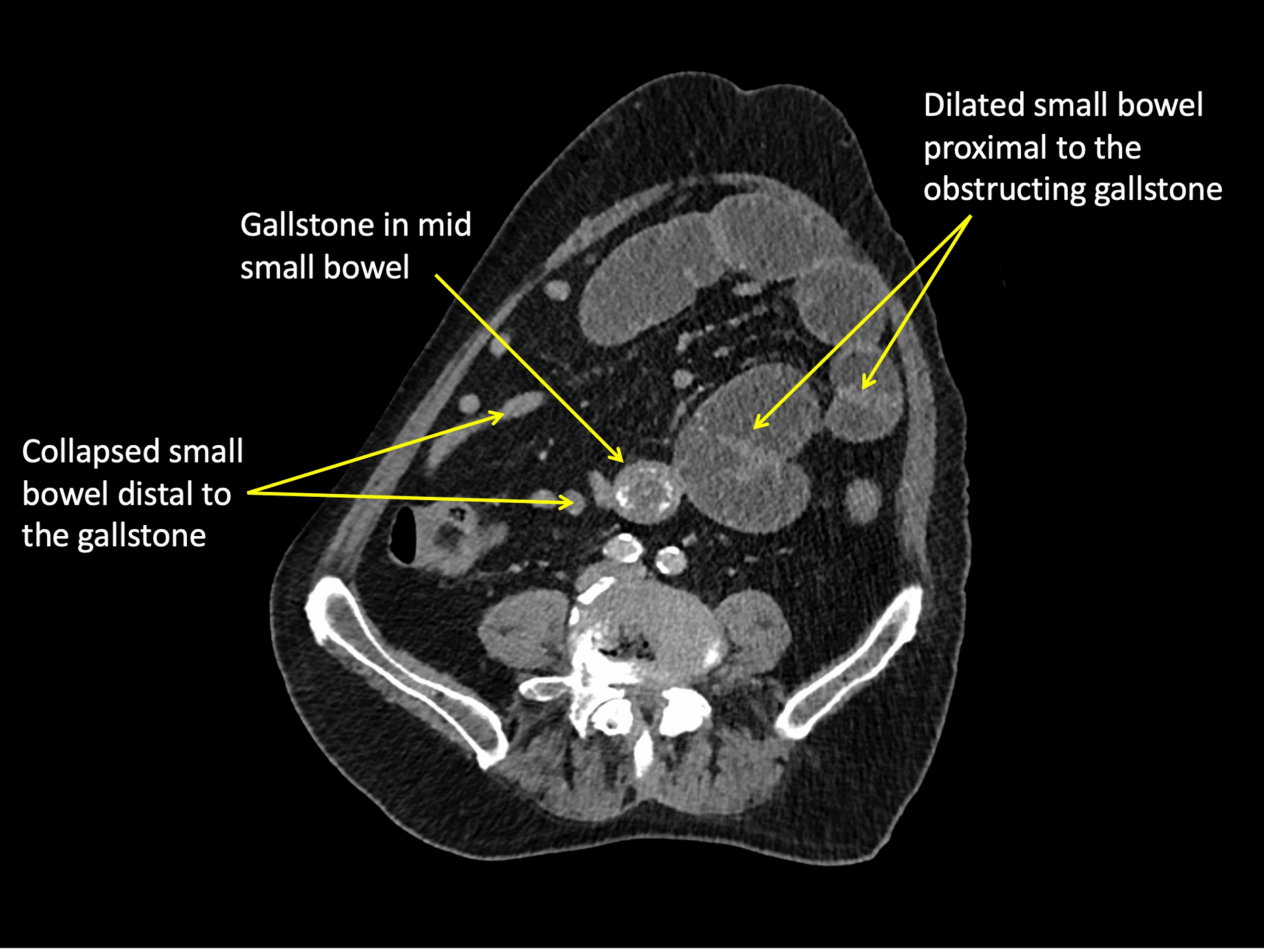
Axial CT showing gall stone in mid small bowel causing proximal small bowel dilatation and collapsed distal loops.

A CT scan showed GSI as a result of an impacted stone in the mid-ileum. The gallbladder was noted to be inflamed and containing stones (Figures 1-2).

Following resuscitation and optimization, the patient was taken for explorative laparotomy, where a transition point in the mid-ileum was identified secondary to an impacted gallstone. The bowel wall at the site of obstruction was healthy and viable, and no migrating stones were felt proximally. Assessment of the gallbladder region showed features of chronic cholecystitis with dense adhesions to the surrounding structures. Considering the age and co-existing morbidities, a decision was taken to relieve the intestinal obstruction by enterolithotomy. An enterotomy was made proximal to the site of impaction, and the obstructing stone was retrieved. The patient progressed well in the immediate postoperative period, tolerating clear oral fluids and opening his bowel. The nasogastric tube was removed, and he was encouraged to take more orally. 

**Figure 3:**
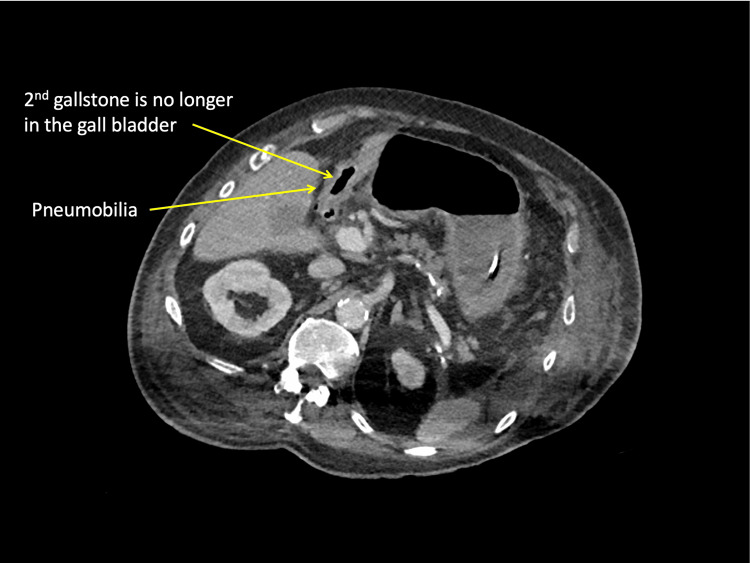
Axial CT showing inflamed thickened gall bladder containing air.

**Figure 4:**
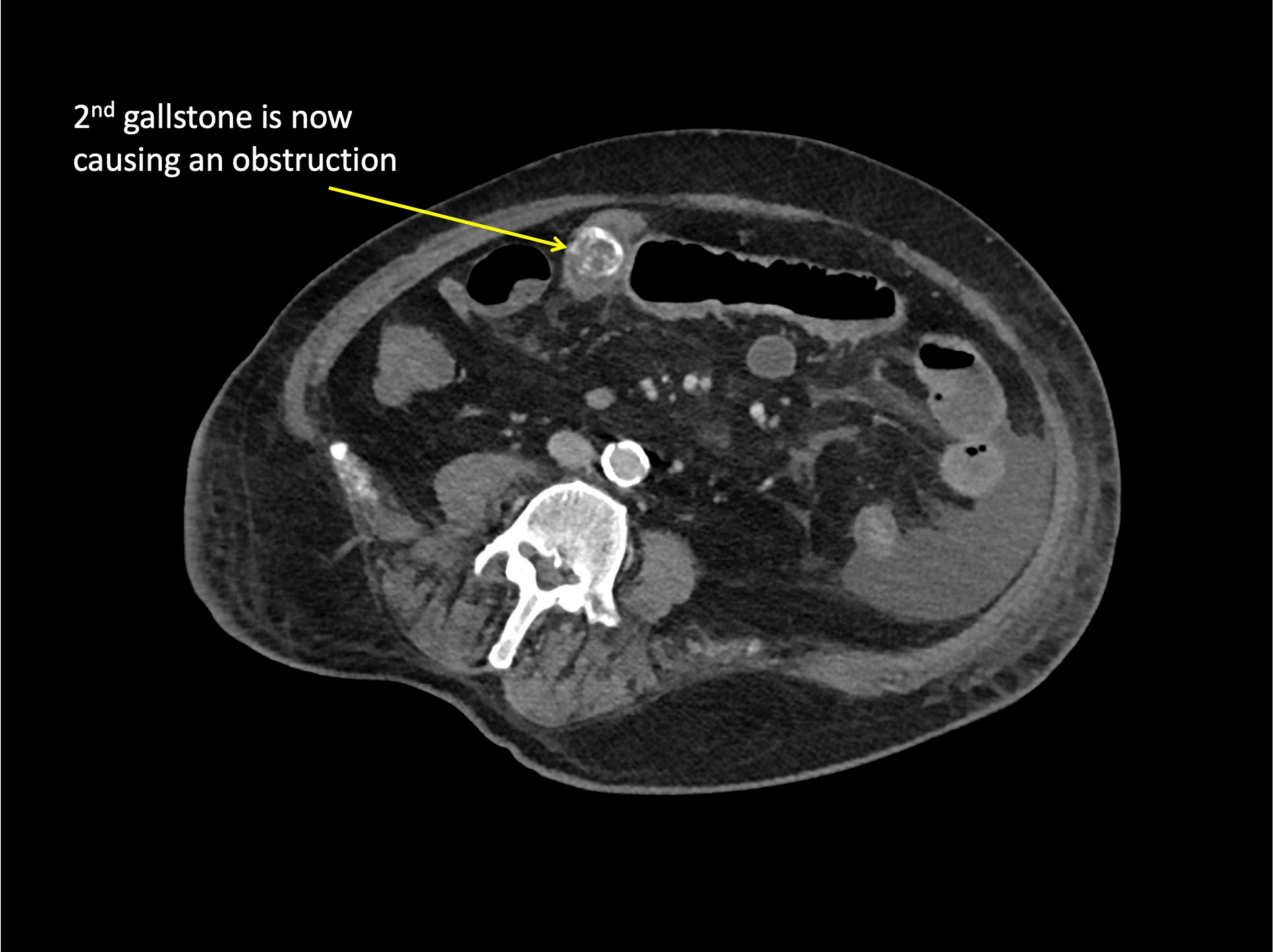
Axial CT showing recurrent gall stone ileus.

On postoperative day 5, the patient appeared unwell with vomiting. Abdominal examination showed a soft, distended abdomen with some tenderness over the incision site. There were no signs of peritonism. Bowel sounds were sluggish, giving a clinical impression of paralytic ileus. The patient did not improve with conservative management. A repeat CT scan showed a recurrent small bowel obstruction secondary to another gallstone (Figures 3-4).

The patient underwent an enterolithotomy with extraction of an impacted stone. Postoperative recovery was uneventful, although prolonged due to the patient's frailty and the nature of his existing illness. 

## Discussion

First described by Bartholini in 1654, GSI is a mechanical obstruction caused by an impacted stone in the lumen of the gastrointestinal tract. It is 4-8 times more common in females than males [8]. About 4000 cases of GSI and 113 cases of recurrent gallstone ileus have been reported in the literature. The incidence is probably high and under-reported. Recurrent gallstone ileus has been reported in about 5-8% of patients following enterolithotomy alone, with the majority of recurrence occurring within 6 weeks to 6 months of index attack [9,10]. Less than 10 cases have been reported where recurrence occurred during index admission [11].

A history of repeated attacks of cholecystitis on a background of cholelithiasis has been thought to contribute to the development of bilio-enteric fistula. The recurrent inflammatory episodes result in the gall bladder adhering to the enteric system, subsequently leading to a bilio-enteric fistula. The stones erode through this fistula into the intestines [6,9,12]. About 80% of stones pass naturally, while stones two centimeters or more in diameter are likely to get impacted, causing bowel obstruction [6,13]. The most common site of obstruction is the terminal ileum in 50-60% of cases [6,14], followed by the jejunum (16-26%), duodenum (3-14%), and colon (3-4%) [10,15]. Colonic obstruction usually occurs if there is a pre-existing pathology such as stricture [16,17]. Bouveret’s syndrome is a rare condition where impaction at the duodenum causes gastric outlet obstruction [6] while Barnard’s syndrome refers to an obstruction in the terminal ileum [15]. Spontaneous closure of the fistula has been reported to be as high as 50% once the stone has passed through the fistula [12]. 

Patients presenting with gallstone ileus or recurrence are usually elderly with poor functional status [13,18]. They may present as an emergency with features of intestinal obstruction or may report vague, nonspecific symptoms such as being unwell, vomiting, confusion, or being "off feet." The mean symptom duration before intervention has been reported to be 4-5 days [15]. It is not uncommon for patients to remain in the ward for days before surgical intervention. Clinical examination may show a distended abdomen and increased bowel sounds. A ‘tumbling phenomenon’ has been described in which there are intermittent symptoms due to a short-lived partial obstruction, which recurs as the stone lodges further distally in the bowel lumen. This phenomenon can delay the diagnosis, which may result in further deterioration, leading to dehydration, shock, sepsis, and peritonitis. In a series of 22 patients with GSI, more than 85% of these patients are of ASA III or IV [19].

Radiological imaging, such as plain radiography, has been found to be of low diagnostic value. The classic Rigler’s triad, characterized by pneumobilia, ectopic gallstone, and small bowel obstruction, is pathognomonic for gallstone ileus when two out of three features are present [20, 21]. Rigler’s triad, however, is only present in less than half of patients with gallstone ileus [22]. Ultrasound scan is useful in assessing the fistula tract, pneumobilia, primary or residual gall stones [23]. However, it is not the preferred imaging modality as a distended fluid-filled abdomen restricts visualization and interpretation. CT scan is considered the gold standard in diagnosis, with a sensitivity of 93% and specificity of 100% [8,24]. Findings consistent with GSI would include features of intestinal obstruction, impacted gallstone, gall bladder wall thickening, and pneumobilia. It is also useful in assessing lesions such as strictures or diverticulae. Gastroduodenoscopy may demonstrate an impacted stone in the duodenum, and therapeutic procedures such as endolithotripsy could be performed [25]. The role of the HIDA scans in assessing the bilio-enteric fistula is limited [26]. 

The treatment option is primarily surgical and tailored according to individual needs. Nonoperative management has a mortality of 26% [10]. An ideal surgical approach with curative intent would be to relieve the obstruction by extracting the obstructing stone, followed by cholecystectomy and repair of the bilio-enteric fistula, all performed in a single setting. This would then prevent recurrent episodes of cholecystitis, cholangitis, and recurrent gallstone ileus. It would also prevent malabsorption and weight loss from a persistent bilio-enteric fistula.

While this approach appears to be ideal, in practicality, this may not be possible as the majority of these patients are physiologically and biochemically compromised at the time of initial presentation. Most of them are elderly and frail, needing optimization prior to surgical intervention. Furthermore, the entire procedure would entail a longer operating time, thus further straining their limited physiological reserves. The technical skills of the operating surgeon are crucial, particularly when the fistula tract involves the common bile duct, as this may require resection. Complications such as bile leak, duodenal leak, residual collections, or strictures can be technically more challenging to manage and may need further surgical or radiological intervention. Reisner had shown a high morbidity of 62% in patients who underwent a radical one-staged procedure compared to those who underwent enterolithotomy alone. Interestingly, the mortality in those groups was similar, approximately 17% with a radical approach and 12% in a single-step procedure [10]. 

Intestinal obstruction is managed with enterolithotomy, while cholelithiasis and bilio-enteric fistula are addressed together with a combined procedure involving cholecystectomy and fistula excision. A bile duct exploration may be needed in some cases. The initial management, therefore, would be enterolithotomy, and the surgeon should judge the suitability of the patient to undergo second-stage surgery. Low-risk patients (ASA Grade I or II ) can undergo both stages in one setting, while high-risk patients may not be suitable, and the biliary procedure can be deferred to a later date [3]. 

Enterolithotomy involves making a longitudinal enterotomy on the anti-mesenteric border proximal to the point of impaction. The stone is milked proximally and removed. Inspection of extracted stone is important as round or faceted stone may indicate the likelihood of multiple stones being present [9, 10]. The enterotomy is closed transversely to minimize the risk of stricture. Nearly half the reported cases of recurrent GSI are due to faceted stones that may have been missed during the index operation [9]. It is, therefore, important to inspect the entire small bowel for migrating or "tumbling" stones. The procedure can be done laparoscopically, although assessment of the obstructed distended bowel along with an increased risk of enteric contents forcefully spilling into the abdominal cavity may make the procedure challenging. Prophylactic antibiotics are administered to minimize the risk of post-operative wound infection. 

While enterolithotomy alone is sufficient for the majority of patients, a small percentage of patients are prone to develop recurrent GSI either during index admission or within a few weeks or months. This cohort poses a challenging decision to the operating surgeon whether to do a simple enterolithotomy or deal with the cause of recurrence, i.e., to do cholecystectomy and repair of fistula. Enterolithotomy is technically less demanding, while single-stage radical surgery requires a longer operating time and a surgeon experienced in biliary tract surgery. As this approach carries higher morbidity and mortality, those favoring entero-lithotomy argue the additional procedure may increase risk in already frail, co-morbid patients. Since the majority of these patients are elderly and compromised during emergency presentation, and more than 90% remain asymptomatic following a simple entero-lithotomy, the overall consensus is to do what is “minimum necessary” for these patients.

A technique of stone extraction from the gall bladder fundus followed by closure of the fundus has been suggested [27]. For patients deemed unfit for surgery, non-surgical treatment such as extracorporeal and electrohydraulic lithotripsy or endoscopic removal has been performed for obstructing stones [28-33]. Despite improvement in surgical technique and peri-operative care, the mortality in these patients is about 6-7% and is 5-10 times higher than other causes of mechanical small bowel obstruction.

In summary, GSI and recurrent gallstone ileus are rare surgical emergencies affecting a high-risk demographic comprising elderly and co-morbid patients. It poses a different set of challenges to the operating surgeon, particularly in deciding the best operative approach to address the emergency situation while being aware of recurrence during index admission itself. The treatment for GSI and recurrent GSI should be surgical unless contraindicated, as mortality in patients undergoing conservative management is high. Enterolithotomy would be the preferred surgical option. Cholecystectomy and repair of bilio-enteric fistula could be considered as an additional procedure in patients with good physiological reserves to prevent recurrence. In these patients, the procedure could be done in two stages; stage one would entail enterolithotomy followed by a second stage involving planned elective cholecystectomy and closure of bilio-enteric fistula [31,34-39]. 

Cholecystectomy could be considered after enterolithotomy in patients who are deemed fit to withstand an additional procedure in the emergency setting. These patients could undergo an elective fistula repair at a later stage. The combined procedure would not, however, preclude recurrent GSI, as migrating stones or residual stones in the CBD can still cause recurrent GSI. In patients who had simple enterolithotomy, early recurrent GSI at index admission may mimic post-operative ileus with resultant diagnostic and management delay. Re-entering a hostile abdomen within a few days following a laparotomy may itself increase morbidity and iatrogenic injury to the bowel or post-op complications in these patients. The recurrence of gallstone ileus may be a strong indicator for definitive management of the fistula, but there is no uniform consensus. The management, therefore, would be enterolithotomy, and the surgeon should judge the suitability of the patient to undergo second-stage surgery.

## Conclusions

This case highlights the importance of keeping a high index of suspicion in patients who fail to progress within the expected time frame in the postoperative period. Following surgery, our patient progressed well initially, but his symptoms recurred again in the immediate postoperative period, giving a clinical impression of paralytic ileus. The CT scan identified recurrent GSI, and the patient had to undergo another surgery to relieve the intestinal obstruction. GSI and its recurrence are rare, affecting high-risk demographics. Early intervention would be in the best interest as delays will further compromise their nutritional, physiological, and biochemical status. Surgical strategy must be individually tailored to consider co-morbidities and general physiological status.
